# *Mycobacterium bovis*: A Model Pathogen at the Interface of Livestock, Wildlife, and Humans

**DOI:** 10.1155/2012/236205

**Published:** 2012-06-10

**Authors:** Mitchell V. Palmer, Tyler C. Thacker, W. Ray Waters, Christian Gortázar, Leigh A. L. Corner

**Affiliations:** ^1^Infectious Bacterial Diseases Research Unit, National Animal Disease Center, Agricultural Research Service, United States Department of Agriculture, Ames, IA 50010, USA; ^2^Instituto de Investigación en Recursos Cinegéticos (CSIC-UCLM-JCCM), Ronda de Toledo s/n, 13005 Ciudad Real, Spain; ^3^School of Veterinary Medicine, University College Dublin, UCD, Dublin 4, Ireland

## Abstract

Complex and dynamic interactions involving domestic animals, wildlife, and humans create environments favorable to the emergence of new diseases, or reemergence of diseases in new host species. Today, reservoirs of *Mycobacterium bovis*, the causative agent of tuberculosis in animals, and sometimes humans, exist in a range of countries and wild animal populations. Free-ranging populations of white-tailed deer in the US, brushtail possum in New Zealand, badger in the Republic of Ireland and the United Kingdom, and wild boar in Spain exemplify established reservoirs of *M. bovis*. Establishment of these reservoirs is the result of factors such as spillover from livestock, translocation of wildlife, supplemental feeding of wildlife, and wildlife population densities beyond normal habitat carrying capacities. As many countries attempt to eradicate *M. bovis* from livestock, efforts are impeded by spillback from wildlife reservoirs. It will not be possible to eradicate this important zoonosis from livestock unless transmission between wildlife and domestic animals is halted. Such an endeavor will require a collaborative effort between agricultural, wildlife, environmental, and political interests.

## 1. Introduction

Zoonotic diseases are responsible for most (60.3%) emergent diseases of humans. Moreover, the preponderance (71.8%) of emerging pathogens are of wildlife origin or have an epidemiologically important wildlife host [1]. The emergence of newly recognized diseases in wildlife is the result of complex, and sometimes unintended, interactions between wildlife, domestic animals, and humans, in terms of host ecology, pathogen, and environment [2, 3]. These interactions include factors such as translocation or introduction of wildlife to new ecosystems, encroachment of human populations on traditional wildlife habitat, artificial feeding of wildlife, and transmission of livestock diseases to wildlife [2]. Wild animals are susceptible to infection with many of the same disease agents that afflict domestic animals and transmission between domestic animals and wildlife can occur in both directions. For veterinarians, diseases in wildlife that are notifiable, eradicated, or near eradication in domestic animals are most problematic. A single case of a reportable disease in livestock can result in serious economic consequences for the producer, public, and government [4].

Transmission of *Mycobacterium bovis* from domestic animals to wildlife (spillover) and subsequent transmission from wildlife back to domestic animals (spillback) is a theme common in several regions of the world attempting eradication of *M. bovis* infection. In most cases, both spillover and spillback have been facilitated by anthropogenic factors such as encroachment on wildlife habitat, animal translocation, or supplemental feeding of wildlife. The scrutiny of wildlife reservoir hosts is essential in control or elimination of *M. bovis* from livestock. Total eradication of any disease is impossible if wildlife maintain a reservoir of infection.

Critical to control of tuberculosis is the understanding of spillover hosts and maintenance hosts. Among spillover hosts, disease does not persist without an external source of reinfection. This external source of infection is often a separate population of susceptible hosts, wild or domestic. In most cases, *M. bovis* was originally introduced by spillover from domestic cattle (maintenance host) to a susceptible wild population (maintenance or spillover host). By definition, disease in spillover hosts will disappear as disease is eliminated from the source of infection. Spillover hosts may be dead end hosts and play no role in disease transmission, but disease may persist for a limited time. In contrast, among maintenance hosts, disease persists without an external source of reinfection. Maintenance hosts may be domestic or wild. There is no sharp demarcation between spillover and maintenance hosts but rather there is a continuum of persistence and transmission efficiency between members of the host populations. For example, the ferret (*Mustela furo*) in New Zealand is a an inefficient spillover host as the disease disappears rapidly from the population due to ineffective intraspecies transmission, but where population density is high, they can act as a maintenance host [5]. Maintenance hosts are critical in disease epidemiology and control because without intervention, disease will persist indefinitely. Hence, the most efficient disease control efforts are aimed at maintenance hosts.

There is general acceptance that among wildlife species the badger (*Meles meles*) in the United Kingdom (UK) and the Republic of Ireland, the brushtail possum (*Trichosurus vulpecula*) in New Zealand, the European wild boar (*Sus scrofa*) in Iberian Peninsula, and the white-tailed deer (*Odocoileus virginianus*) in Michigan, United States (US) represent true maintenance hosts and a source of infection for other species. These maintenance host reservoirs have in common, high population density, and continuous interspecies interaction at the wildlife-domestic animal interface, both of which facilitate disease persistence [6]. Host species alone does not necessarily designate spillover host or maintenance host assignation. In one ecosystem, a particular species may act as maintenance host (i.e., white-tailed deer in Michigan and wild boar in the Iberian Peninsula) [7, 8] while in another ecosystem the same species may act as a spillover host (i.e., white-tailed deer in Minnesota, US and feral pigs in New Zealand and Australia) [6, 9–11]. These differing roles are likely the result of many factors including animal density, environment, and contrasting agricultural and cultural practices.

In the early part of the 20th century, there were large numbers of tuberculous cattle in industrialized regions of North America, Europe, and Australia. Often an association was made between the number of *M. bovis*-infected humans and the prevalence of tuberculosis in the local cattle population. Infected cattle were generally considered the source of human infection with *M. bovis*; transmission being through ingestion of unpasteurized dairy products [12, 13]. Additionally, abattoir workers and veterinarians were infected during slaughter or postmortem examination of cattle [14–16]. More recently, exposure to tuberculous elk (*Cervus canadensis*), white-tailed deer, and possums has resulted in human infection [17–20]. In developed countries, mandatory pasteurization of milk combined with tuberculin testing and slaughter of infected cattle resulted in dramatic declines in the incidence of human tuberculosis due to *M. bovis*. Notwithstanding, in 1995, it was estimated that worldwide 50 million cattle were infected with *M. bovis,* at a cost to the agricultural community of US $3-4 billion per annum [21]. In developing countries, *M. bovis* infection is still widespread, in both cattle and humans. Even in developed countries, successful eradication of disease from livestock is hampered by the presence of wildlife reservoirs of *M. bovis*. In general, countries with a wildlife reservoir of *M. bovis* have not been able to eradicate *M. bovis* infection from livestock. The following examples illustrate the complex interaction of wildlife, domestic animal, and human factors in the creation and maintenance of wildlife reservoirs of tuberculosis.

## 2. White-Tailed Deer in the United States—Supplemental Feeding of Wildlife

Prior to 1994, there had been isolated case reports of tuberculosis in white-tailed deer in the US [22–25]. All reports involved 1 or 2 animals and were seen in captive deer, hunter-killed deer, or cases of accidental death. At the time, it was postulated that *M. bovis* had spilled over from tuberculous livestock in the region; however, no followup surveys were conducted and no strain comparisons were made to confirm such a hypothesis. In 1975, a free-ranging white-tailed deer in northern Michigan was diagnosed with tuberculosis due to *M. bovis *[26]. The tuberculous white-tailed deer was thought to be an anomaly and no followup surveys of free-ranging deer were conducted. Meanwhile, Michigan was granted TB-free status by the US Department of Agriculture (USDA) in 1979.

In 1994, a hunter-killed white-tailed deer was identified with tuberculosis due to *M. bovis*. This deer was found only 13 km from the site where the tuberculous deer had been identified in 1975. Subsequent surveys identified a focus of *M. bovis* infection in free-ranging white-tailed deer in northeast Michigan [26]. This represented the first known reservoir of *M. bovis* in free-living wildlife in the US and the first known epizootic of tuberculosis in white-tailed deer in the world. Several factors are thought to have contributed to the establishment and persistence of *M. bovis* in Michigan white-tailed deer. It is postulated that *M. bovis* was transmitted from cattle to deer during the early to mid 1900 s when the prevalence of *M. bovis* in Michigan cattle was high [27]. Statistical models estimate that spillover from cattle to deer occurred around 1955 [28]. During this same period, Michigan's deer population was steadily increasing beyond normal habitat carrying capacity. In 1930 there were an estimated 592,000 deer in Michigan and by 1998, the number of deer had grown to over 1.7 million with focal concentrations of 19 to 23 deer per km^2^. Regions of highest deer density were later found to be the center of the current tuberculosis outbreak [26, 29, 30]. Transmission and maintenance of *M. bovis* among deer in Michigan was facilitated, not only by high deer density, but also by the common practice of long-term winter-feeding of large volumes of sugar beets, carrots, corn, apples, pumpkins, and pelleted feed to deer by the public. Supplemental feeding was intended to decrease winter mortality and prevent migration in order to preserve high deer numbers for hunting purposes [26]. High deer density, combined with prolonged crowding of deer around feeding sites provided opportunity for deer to deer contact and enhanced transmission of tuberculosis [31]. Epidemiologically, supplemental feeding has been documented as a contributing factor to *M. bovis* infection in deer [29]. Specific factors associated with increasing risk of tuberculosis were location of a feeding site near hardwood forest, number of deer fed per year, presence of other nearby feeding sites, and the quantity of grain, fruits or vegetables fed. DNA fingerprinting through restriction fragment length polymorphism (RFLP) analysis of *M. bovis* isolates from Michigan white-tailed deer showed that the majority of deer were infected with a common strain of *M. bovis* suggesting a single source of infection [32]. By 2010, over 188,000 deer had been tested by gross necropsy, bacteriologic culture, and histopathology, and of these, 687 confirmed cases of *M. bovis* infection had been identified in 12 counties in northern Michigan.

### 2.1. Pathology

Tuberculous white-tailed deer most commonly develop lesions in retropharyngeal lymph nodes, followed by the lung and associated lymph nodes [26, 33, 34]. Similar to other species of deer, lesions may grossly resemble abscesses due to other organisms making differential diagnosis important. Unlike red deer (*Cervus elaphus*), elk (*Cervus canadensis*), and fallow deer (*Dama dama*), draining fistulae from superficial lymph node lesions have not been reported in white-tailed deer [35–38]. In these other species, such lesions may be important in disease transmission.

Microscopically, lesions consist of foci of caseous necrosis with or without mineralization, surrounded by infiltrates of epithelioid macrophages, lymphocytes, and Langhan's type multinucleated giant cells. Lesions are often surrounded by variable amounts of fibrous connective tissue with low numbers of acid-fast bacilli (AFB) present within the caseum, macrophages, or multinucleated giant cells. Microscopically, lesions in white-tailed deer are similar to those seen in cattle, although lesions in cattle are generally surrounded by greater amounts of fibrous connective tissue.

### 2.2. The Role of Artificial Feeding of Wildlife

Artificial feeding is broadly defined as placing natural or artificial food into the environment that supplements food in the natural home range of a given wildlife species. Both supplemental feeding and baiting (use of food as an attractant for hunting purposes) of wildlife have been associated with increased transmission of infectious diseases such as tuberculosis and chronic wasting disease [39]. Contact can be direct through physical (nose-to-nose) contact or transmission through infectious aerosolized respiratory droplets, or indirect as occurs when two animals share the same feed or feeding site. Although increased potential for disease transmission is perhaps of greatest concern, feeding and baiting can also disrupt animal movement patterns, spatial distribution, social structure, and result in habitat degradation [40]. Stress from overcrowding around feeding sites can negatively affect immune protection of individual animals, exacerbating disease and increasing the likelihood of disease transmission.

### 2.3. Interspecies Transmission Including Zoonotic Potential

The presence of *M. bovis* in wildlife is not only detrimental to the health of the wildlife population but also represents a serious threat to livestock and a risk to human health. Over 50 *M. bovis*-infected cattle herds have been identified in Michigan since the identification of tuberculosis in white-tailed deer in 1994. RFLP analyses suggest that cattle, deer, and other wildlife are infected with a common strain of *M. bovis*. Cattle probably become infected through direct or indirect contact with free-ranging white-tailed deer [32]. By 2010, estimates suggested the overall cost to Michigan of the presence of *M. bovis* in deer and cattle had been greater than US $100 million [41]. Surveys of carnivores and omnivores in Michigan have confirmed spillover of *M. bovis* infection to coyotes (*Canis latrans*), bobcats (*Felis rufus*), red foxes (*Vulpes vulpes*), black bears (*Ursus americanus*), opossums (*Didelphis virginiana*), raccoons (*Procyon lotor*), and domestic cats (*Felis catus*) [42–44]. Non-deer wildlife are likely to have been infected through scavenging of dead deer carcasses. Infection in nondeer wildlife is characterized by limited lesion development suggesting that they are dead-end spillover hosts and unimportant in maintenance of the epizootic in deer or transmission to other susceptible hosts [45, 46].

In tuberculous humans, aerosol transmission via respiratory secretions containing *Mycobacterium tuberculosis* is the primary means of human-to-human spread. Minute (<5 *μ*m) aerosolized droplets known as droplet nuclei can be generated by talking or coughing [47, 48]. Such nuclei remain airborne for prolonged periods while larger droplets quickly come to rest within a short distance of the host. Some droplet nuclei carry *M. tuberculosis* and once inhaled pass deep in to the bronchi and bronchioles where they can establish infection and initiate the disease process. Both aerosol and oral transmission of *M. bovis* between deer can occur as deer congregate around artificial feeding sites. One study found that *M. bovis*-infected deer were more closely related genetically, than noninfected deer, suggesting that contact within family groups was important in disease transmission [49]. Indeed, deer in family groups are more likely to share feed from the same sources, participate in mutual grooming, and spend time within distances favorable to aerosol transmission.

Aerosol transmission between deer and cattle is less likely to occur as deer are seldom in close proximity to cattle. One study on deer-cattle interactions, within the TB-endemic zone of Michigan, found direct deer-cattle interactions (deer within 5 m of cattle) to be exceedingly rare; however, deer were commonly seen in feed storage areas eating out of hay racks and feeding troughs [50]. Accordingly, most deer to cattle transmission is believed to be indirect through sharing of feed. White-tailed deer experimentally infected with *M. bovis* shed tubercle bacilli in saliva and nasal secretions [51, 52]. Research shows that experimentally infected deer can transmit *M. bovis* to other deer or cattle through both direct (cohoused) and indirect contact such as sharing of feed with no opportunity for direct contact or aerosol transmission [31, 52, 53]. Feed contaminated with saliva and nasal secretions containing *M. bovis* can be a source of infection for other animals.

*Mycobacterium bovis* is relatively resistant to environmental factors and under appropriate conditions (e.g., cool and protected from sunlight) may persist in the environment for weeks or months, prolonging the likelihood of transmission by ingestion [54–58]. A study on environmental survivability of *M. bovis* under natural weather conditions in Michigan found *M. bovis* survived up to 88 days in soil, 58 days in water and hay and 43 days on corn [55]. Although capable of surviving for many weeks in the environment, the risk from environmental *M. bovis* is mitigated by the location of the bacilli in soil or water making the tubercle bacilli less accessible to hosts. Survival on feedstuffs commonly used as supplemental feeds provides a more conceivable route of indirect transmission. The dose required for indirect transmission through the sharing of feedstuffs is unclear, but is likely higher than that required for transmission through direct contact (nose to nose) or aerosol transmission. In utero transmission has not been documented in white-tailed deer; however, potential transmission from doe to nursing fawns has been suggested experimentally, with 3 of 5 fawns infected through the consumption of milk containing 1 × 10^4^ colony forming units (CFU) of *M. bovis*. The frequency of such doe to nursing fawn transmission in nature is likely low and probably not important in the maintenance of disease as mammary gland lesions in naturally infected deer have been rarely reported [59, 60].

Epidemiologic modeling suggests a 2-stage model of transmission within deer populations. Stage 1 involves transmission within matriarchal family groups, allowing disease to persist in the population at a low level [30]. Family groups consisting of a matriarchal doe and several generations of her daughters and their fawns characterize the social structure of white-tailed deer. Fawns from the previous year leave the dam when she nears parturition in spring. Yearling does often rejoin their dam and her fawns in the fall. Stage 2 involves both supplemental feeding, with resultant increased deer density, and dispersal of male fawns to join male groups that travel together at all times except during breeding season [30]. Higher disease prevalence has been observed in adult male deer [8]. Shifting group membership by male deer results in temporary association with several different groups and increased contact with numerous susceptible animals.

Although *M. bovis* is a recognized zoonotic agent, no change in incidence of *M. bovis* infections in Michigan's human population has been detected since the epizootic was recognized [19]. However, two cases of *M. bovis* infection in humans have been linked to *M. bovis* found in free-ranging deer [20]. One of the two cases was cutaneous tuberculosis in a hunter, the result of an injury sustained during field dressing of a tuberculous white-tailed deer. In spite of the paucity of cases, there are potential risks as hunters are exposed to *M. bovis* during the field dressing of deer or the consumption of undercooked venison products. Michigan's Departments of Community Health, Natural Resources, and Agriculture have worked cooperatively to educate hunters, farmers, and Michigan residents on the identification of tuberculosis in deer, recommended personal protective measures, and the importance of thorough cooking of venison prior to consumption [19].

### 2.4. Disease Control Effort

In Michigan, wildlife and domestic animal health authorities have adopted control measures that (1) reduce deer density and population through increased hunting, (2) restrict or eliminate supplemental feeding of deer, and (3) monitor both wildlife and livestock through hunter-killed deer surveys, carnivore and omnivore surveillance, and whole-herd tuberculin testing of cattle. These control measures appear to have succeeded in preventing an increase in prevalence and geographic spread of tuberculosis in white-tailed deer in Michigan. In 1998, supplemental feeding was banned in counties where tuberculous deer had been identified. Enforcement has been problematic and universal compliance has not been achieved. Public and political pressures have resulted in an easing of prohibitions that allow baiting in previously restricted areas. Deer numbers have been reduced by 50% in the endemic areas through increased hunting pressure and unlimited harvesting of female deer. However, progress towards eradication will likely require additional actions and more time. Epidemiological modeling suggests that further decreases in deer density and a strictly enforced ban on supplemental feeding will be required to eradicate *M. bovis* from Michigan wildlife and cattle.

### 2.5. Vaccination

*Mycobacterium bovis* strain bacilli Calmette-Guerin (BCG) has been used as a vaccine and showed protection in cases of naturally occurring tuberculosis in sika deer (*Cervus nippon*) [61], and in experimental infections of red deer [62, 63]. Using experimentally infected white-tailed deer, both BCG strains Pasteur and Danish provided protection in the form of decreased lesion severity [64–66]. In vaccinates, there were fewer, smaller, and less extensive lesions compared to nonvaccinates. Lesions in vaccinates were characterized by less necrosis and fewer AFB than in nonvaccinates. One study showed that oral vaccination provides equivalent protection when compared to subcutaneous vaccination [64].

BCG can persist in tissues of vaccinated deer. Studies to examine BCG persistence demonstrate that after oral or subcutaneous administration, BCG persisted in tissues for up to 3 and 9 months respectively [67]. Shedding of BCG by vaccinates was assumed to have occurred as nonvaccinates sharing the same pen became infected with BCG [65, 66, 68]. Vaccine shedding to nonvaccinated animals has not been described in BCG vaccination studies in cattle or red deer. However, studies to examine transmission from vaccinated deer to nonvaccinated cattle through indirect contact have been unsuccessful [68].

### 2.6. The Minnesota Experience

In 1971, Minnesota was considered free of bovine tuberculosis and was granted TB-free status by the USDA. However, in 2005, a beef cow infected with *M. bovis* was discovered through meat inspection surveillance [6]. The cow originated from northwestern Minnesota. Testing of remaining cattle in the herd revealed a prevalence of 1.2%. Epidemiological investigations identified 4 other herds in the region with tuberculous cattle. The discovery of bovine tuberculosis prompted surveillance of local free-ranging deer. Harvesting of 474 deer yielded 1 deer infected with *M. bovis*. The response by Minnesota animal health and wildlife authorities was aggressive and included statewide testing of all cattle, dramatically decreasing deer density in the region through increased removal by hunters, landowners, and government officials, removal of many of the cattle in the area through a voluntary buy-out program, and fencing of feeding areas on remaining farms with cattle herds. Over 6200 head of cattle were removed from the region at a cost of US $4.6 million. Between 2005 and 2009, *M. bovis* was found in 27 deer and 12 cattle herds [6]. In 2010, no *M. bovis*-infected deer or cattle herds were found within the state, and as of November 2011, Minnesota regained TB-free status by the USDA. The effort at preventing the establishment of a wildlife reservoir of *M. bovis* in Minnesota's white-tailed deer was aggressive, but not without cost. The costs to USDA were estimated at US $70 million, Minnesota Board of Animal Health US $12.5 million, and Minnesota Department of Natural Resources US $3.5 million.

## 3. Badgers in the United Kingdom and Ireland—Spillover and Spillback

*Mycobacterium bovis *is endemic among badgers in southwest England, south Wales, and Ireland. It is hypothesized that badgers are a source of infection for cattle and responsible for an increase in tuberculosis among domestic cattle herds in the UK.* Mycobacterium bovis* was first isolated from badgers in Switzerland in 1957 [69]. In 1971, the first tuberculous badger was identified in England [70] and in 1975 the first infected badger was reported in Ireland [71]. It is believed that badgers became infected with *M. bovis* by spillover of infection from cattle during the late 19th and early 20th centuries when a large percentage of British and Irish cattle were infected with *M. bovis.* By the 1970s, bovine tuberculosis had been removed from large areas of the UK, and animal health authorities anticipated eradication. In 1981, the Wildlife and Countryside Act provided protection of badger populations in the UK, and in Ireland protection was granted in 1976. Protection has resulted in a large increase in badger populations in both countries. Over the past decades, the UK has experienced a rising incidence of bovine tuberculosis but herd incidence rates have remained constant in Ireland. In 1998, fewer than 6% of herds in the UK were under movement restriction due to bovine tuberculosis, this figure had increased to more than 13% by 2010 [72].

### 3.1. Badger Ecology

The badger is a mustelid, a family of carnivorous mammals. They are nocturnal and live in social groups of mixed ages and sexes in underground setts. Setts are elaborate structures of multiple interconnecting tunnels and nest chambers with numerous entrances that can be found throughout the territory of a social group [73]. They are used for resting, breeding, protection from predators, shelter from harsh weather, and emergency refuge [73]. In areas of high population density, as in southwest England, social groups may consist of 8–20 individuals [74, 75] but in low density areas, as in Ireland, social groups are smaller and consist of only 2 to 3 individuals [76]. Social groups are territorial. Territories are well defined and stable over time in areas of high population density, as in southwest England [77], but are less clearly demarcated in low-density areas [78, 79]. There is a constant level of intergroup mingling but during the breeding season territories are fiercely defended resulting in high levels of intergroup aggression. In areas of low density, there appears to be proportionately more intergroup mingling [78].

The badger's natural habitat is such that it lives on or near pastures used by cattle where it seeks carrion and digs for earthworms, frogs, and insect larvae [80]. Setts provide ideal conditions for the spread of respiratory diseases. In southwest England, where the highest density of badgers is found, badger density can be as high as 25.3 adults per km^2^; but in Ireland the density is 1-2 adults per km^2^ [81]. There appears to be no direct correlation between badger density and the prevalence of *M. bovis* infection among badgers [75, 82].

### 3.2. Pathology

Tuberculosis is a chronic infection that progresses slowly with infected badgers maintaining a normal life expectancy. Badgers are very susceptible to infection with *M. bovis*, with infection established by endobronchial instillation of doses below 10 CFU, yet they are able to control infection with higher doses (*∼*10^4^ CFU) [83]. Latent tuberculosis infection, that is, infection in the absence of gross lesions, is found in 50% to 80% of naturally infected badgers in wild populations [84]. Both naturally infected and experimentally infected badgers have few sites of infection but lesions can be found in a wide range of anatomical locations. In badgers infected by natural transmission, the most frequent sites of infection are the lungs, lung-associated lymph nodes, and medial retropharyngeal and axillary lymph nodes, while renal infection is infrequent [85]. The frequency of infection and the anatomical sites affected is considerably greater than can be appreciated from the distribution of gross lesions [86, 87]. Tuberculous granulomas in badgers are composed predominantly of epithelioid cells, macrophages, and few lymphocytes. They are highly cellular and proliferative, with little necrosis, mineralization, or fibrosis and have the same general appearance in all tissues [88]. The lesions are interstitial and expansive, compressing surrounding parenchyma. Histological features that are characteristic of tuberculosis in other species, such as caseation, fibrous tissue encapsulation, cavitation, abscessation, or Langhan's multinucleated giant cells are not seen in badgers. The histopathology of tuberculosis in badgers resembles that seen in other carnivores [89].

### 3.3. Intraspecies and Interspecies Transmission Including Zoonotic Potential

Badger to badger transmission is most likely through aerosols and to a lesser extent through bite wounds [82]. Experimental studies demonstrate that badgers can transmit *M. bovis* to cattle [90]. The exact route of transmission is unclear; nevertheless, based on excretion patterns it is probably via infectious aerosols. Infected badgers shed *M. bovis* in respiratory secretions and exudates from draining superficial lesions [87]. Shedding in urine and feces only occurs in the small proportion of badgers with generalized disease [85]. As infected badgers have been observed to live for 3-4 years after shedding of *M. bovis* was first detected, they are an excellent maintenance host of *M. bovis* with even mildly infected badgers constituting an ongoing risk. [91]. It is suggested that cattle may become infected by inhalation and less likely ingestion of fodder contaminated with infected badger sputum, urine, feces, or exudates from superficial lesions [92]. Badgers mark territory boundaries at localized areas used for urination and defecation known as latrines and likewise mark travel pathways with urine [93]. Both latrines and pathways are generally accessible to cattle making them areas of risk for cattle. High doses of *M. bovis* are required to infect cattle by ingestion; however, excretion of high numbers of tubercle bacilli in urine and feces is uncommon in badgers [85]. Experimentally, calves have been infected when housed with experimentally infected, as well as naturally infected badgers [90]. Five of nine calves became infected after being housed with infected badgers that were shedding *M. bovis*; exposure was for periods of 6 to 12 months and all infected calves had lung and thoracic lymph node lesions with one having additional lesions in the medial retropharyngeal and mesenteric lymph nodes. Epidemiological studies show that areas with the greatest density of badgers have the highest incidence of tuberculosis among cattle [70, 82, 94]. Consequently, in Ireland, cattle have been shown to be effective sentinels for tuberculosis in badgers [95].

Recently, since the resurgence of disease in the UK, the first documented cases of spillover of *M. bovis* from animals to humans were reported [96]. Two siblings residing on a farm were diagnosed with tuberculosis due to *M. bovis*. Cattle on the farm had also been diagnosed with *M. bovis*. The cattle isolate was indistinguishable from the isolates from the 2 siblings when examined by RFLP analysis, spacer oligonucleotide typing (spoligotyping), and variable number tandem repeat (VNTR) analysis, suggesting transmission between cattle and humans. Moreover, the farm supported a large badger population where *M. bovis* infection had been previously diagnosed. It is suggested, although not proven, that cattle became infected through contact with badgers and that humans became infected through contact with cattle.

### 3.4. Disease Control Effort

Badgers are an ideal host for *M. bovis*; infection in populations is usually endemic, tuberculosis kills few badgers, and their deaths do not significantly perturb population density nor the size and structure of social groups. Badgers may survive for long periods while suffering from overt disease; however, the majority of infected badgers remain clinically healthy, with a high proportion (50–80%) having latent infection. Tuberculous females often continue to produce cubs [82]. The removal of infected badger populations from cattle farming areas has resulted in a decline in bovine tuberculosis in cattle. However, reports of the efficacy of culling in decreasing the risk of spread of infection from badgers to cattle differ between those of Ireland and the UK.

Evaluation of badger culling trials conducted in the UK from the 1970s to the mid-1990s failed to provide clear outcomes due to their small size and lack of controls [94]. In the UK, following the first suggested links between badgers and bovine tuberculosis, farmers were allowed to cull all badgers in individual setts. This type of proactive culling was later replaced with a strategy to identify and remove clusters of infected badgers (reactive culling). Over a 6-year period, more than 20,000 badgers were culled in an attempt to control escalating rates of bovine tuberculosis [97]. From 1986 to 1998, reactive culling occurred only on land where tuberculin-test-positive cattle were present [98]. Increasing spread of tuberculosis in cattle led to the suspension of badger culling in England and Wales and the appointment of an independent advisory committee, the Independent Study Group on Cattle TB (ISG), with the broad charge of examining the role of badgers in the epidemiology of tuberculosis in animals and to investigate options for badger control [99]. In 1998, a large experiment was implemented, under the direction of the ISG, known as the Randomized Badger Culling Trial (RBCT). The RBCT was designed to determine the role of badgers as a reservoir of *M. bovis* and to compare the effects of three different control strategies; no culling of badgers, localized selective culling of badgers in response to identified cases of tuberculosis in cattle (reactive culling), and removal of all badgers across entire trial areas (proactive culling). The trial clearly demonstrated that infected badgers were a reservoir of infection for cattle [100]. The reactive culling component of the RBCT was prematurely curtailed when analysis of data from the first few years suggested that reactive culling had increased disease risk in cattle herds [98]. Little useful results have been generated from the reactive culling component of the RBCT and the analysis leading to the cessation of reactive culling has been severely criticized [101, 102]. In contrast, after 5 years of proactive culling, there was a 23% reduction in the incidence of cattle tuberculin reactors inside the culling area and an ongoing beneficial effect, with a 54% decrease 1-2 years after the last proactive cull [99].

In Ireland, a national bovine tuberculosis eradication policy was initiated in 1954. Progress in the form of decreasing prevalence was good through the mid-1960s. In the first 11 years of the program, overall prevalence of bovine tuberculosis decreased from 17% to 0.3% [103]. As in England and Wales, tuberculosis is endemic in the badger population in Ireland and the *M. bovis* strains from badgers and cattle are identical by RFLP and spoligotyping analysis [104]. Although badgers were given legal protected status Ireland in 1976, proactive culling of badgers for research and focal (reactive) culling are allowed through licenses granted by the National Parks and Wildlife Service. In Ireland, focal (reactive) culling in response to a herd outbreak is undertaken only after an epidemiological investigation has eliminated all other sources of infection [105]. Culling remains an interim strategy while research on alternative control options are investigated [106]. The case for culling is supported by research conducted in Ireland. Epidemiological reports of tuberculin reactors in cattle in 1988 found evidence of badger involvement in 14% of cases [107]. That infected badgers pose a significant risk to cattle has been shown in two studies, the East Offaly study (1988) and the “Four Areas” (1997–2002) study [103]. In both studies, proactive badger removal was shown to significantly decrease herd incidence of bovine tuberculosis. The East Offaly study showed that control herds, where no badgers were removed, were twice as likely to have cattle movement restricted due to bovine tuberculosis than herds in the badger removal areas [107]. The Four Areas study was conducted between 1997 and 2002 in matched removal and reference areas (average area 245 km^2^), in four counties with differing agricultural land types and farming practices. Proactive badger culling was intensive and thorough in removal areas, but in the reference areas, reactive culling was only done in response to a severe tuberculosis outbreak in cattle. During the study, the odds and hazard ratios of a herd movement restriction due to bovine tuberculosis in the removal areas were significantly lower than in matched reference areas [103]. Reactive culling has also been shown to have a broader beneficial impact than just protection of the herd at the center of the culling. In County Laois between 1989 and 2005, reactive badger removal had a significant beneficial impact on the risk of future outbreaks in herds surrounding the area where badgers were removed [108].

Why were the experiences in the Republic of Ireland and the UK so different? The seemingly conflicting findings from the badger culling studies in Ireland and England may be partially due to the presence of geographical barriers in the Irish study that could have impeded badger movement, such as coastline, sea inlets, mountain ranges and rivers, differences in badger density, and trapping efficacy [97, 103]. As badger immigration was noted as a compounding variable in the East Offaly study, trial locations in the “Four Areas” study were intentionally selected to maximize natural boundaries (e.g., coast, rivers, mountains) so as to minimize the effect of badger immigration. Indeed, the success of the Irish “Four Areas” study may have resulted from a combination of low badger population density, limited immigration, and effective badger removal over a geographical area larger than the RBCT [97]. In the “Four Areas” study, researchers sought to achieve as complete removal as possible over a large area, and to sustain this effort throughout the 5-year study period [103].

Badgers have complex social structures, the stability of which varies depending on population density [78]. Examination of the RBCT culling areas, which support some of the highest badger densities ever recorded, revealed that culling resulted in social restructuring and increased home range of remaining badgers. Increased ranging behavior likely resulted in increased contact with other badgers, as well as cattle [109]. In the UK studies, social restructuring and increased badger movements have been correlated with an increased incidence of *M. bovis* infection among badger populations [75]. In the low-density Irish badger population, culling may have resulted in less social disruption.

One theoretically effective means of preventing transmission of infection is segregation of cattle from badgers. Accordingly, cattle husbandry practices aimed at separating cattle and badgers have been proposed as a means of tuberculosis control. Exclusion of badgers from cattle housing and feeding areas is a feasible control measure. Keeping cattle away from badger setts, urination trails, and latrines may be beneficial, but keeping badgers away from cattle at pasture would be expensive and result in disruption of normal badger behavior patterns [110]. Whereas public attitudes in the UK are not in favor of badger culling and surveys show public preference of conservation and animal welfare over disease control, in Ireland, culling is accepted as an interim policy and is under continuing review by the National Parks and Wildlife Service.

### 3.5. Vaccination

Complete removal of any wildlife reservoir of infection would be extremely difficult, unethical, and would contravene commitments by the UK and Ireland to the Bern Convention on the Conservation of European Wildlife, which promote responsible conservation of native species. In the long term, most believe the best prospect for control of bovine tuberculosis in the UK and Ireland is through vaccination of cattle or wildlife, combined with improved diagnostic tests to distinguish vaccinated from infected cattle [94, 106]. BCG is the most likely candidate for a badger tuberculosis vaccine as it induces protection after being administered by subcutaneous, conjunctival, intranasal, and intramuscular routes [111]. The first demonstration of BCG vaccine-induced protection in badgers was reported in 1988 using the intradermal route of administration [112]. Vaccinates lived longer and shed fewer tubercle bacilli than the nonvaccinates. BCG has also been found to be protective in badgers when delivered by either mucosal [111] or oral [88] routes [113, 114]. For BCG vaccination of wildlife, an oral bait is the most practical means of delivery [115]. For oral administration, BCG was encapsulated in a lipid matrix that provided protection of the bacilli from the lethal effects of gastric secretions [116]. In a UK field trial, BCG vaccinated badgers had a lower rate of seroconversion (a useful indicator of *M. bovis *infection in badgers) than controls [113]. Testing of the lipid encapsulated oral BCG vaccine in a large-scale field trial is currently underway in Ireland [117], while in March 2010, the UK veterinary medicines licensing body granted approval for use of *M. bovis* BCG strain Danish 1331, in badgers (intramuscular use only) [99], where it will be used in a vaccine deployment project (http://www.defra.gov.uk/fera/bvdp/).

### 3.6. Other Wildlife

In 2004, the results were released of a study to examine numerous species of wildlife in the UK for tuberculosis. Over 4700 animal carcasses were examined and tissue samples processed for isolation of *M. bovis*. Infection was confirmed in foxes, stoat (*Mustela erminea*), polecat (*Mustela putorius*), common shrew (*Sorex araneus*), yellow-necked mouse (*Apodemus flavicollis*), wood mouse (*Apodemus sylvaticus*), field vole (*Microtus agrestis*), grey squirrel (*Sciurus carolinensis*), roe deer (*Capreolus capreolus*), red deer, fallow deer, and muntjac deer (*Muntiacus reevesi*). Sample size varied widely, but the highest prevalence was seen in polecats (4.2% of 24), stoats (3.9% of 78), foxes (3.2% of 756), yellow-necked mouse (2.8% of 36), common shrew (2.4% of 41), field vole (1.5% of 67), roe deer (1.0% of 885), red deer (1.0% of 196), fallow deer (4.4% of 504), and muntjac deer (5.2% of 58). A qualitative risk assessment based on prevalence, likelihood of excretion, likelihood of contact with cattle, and animal biomass identified fallow deer and red deer as the highest risk for transmission of *M. bovis* to cattle [118]. This survey demonstrates that deer may pose a significant risk to cattle, especially in regions where deer density is high. However, with regional tuberculosis, prevalence as high as 20.5% in badgers, badgers remain a primary concern for tuberculosis control in the UK. Recently, *M. bovis* was reported in a free-ranging wild boar in the UK. Boar in the wild have not been present in England for several centuries; however, escapes from captive facilities and deliberate releases have resulted in small feral populations. *Mycobacterium bovis* has been isolated from captive boar on only 3 occasions since 1997.

## 4. Brushtail Possum in New Zealand—Wildlife Introduction and Translocation

Although in New Zealand *M. bovis* infection has been found in 14 different domestic and wild animal species, the most significant wild maintenance host is the brushtail possum with deer species, particularly red deer, and ferrets being spillover hosts [5]. Prior to arrival of the first humans, the only mammals present in New Zealand were 2 species of bats [119]. Early European settlers introduced cattle approximately 200 years ago and these settlers were responsible for clearing large areas of forest to accommodate pastoral farming. Europeans introduced 31 other mammal species to New Zealand including brushtail possums, ferrets, and seven deer species [120].

Brushtail possums were first introduced to New Zealand from Australia in the mid-19th century to establish a fur trade. Between 1837 and 1922, over 30 groups of possums were imported, maintained in captivity for breeding, and released at over 160 different sites around New Zealand [119]. Lack of natural predators combined with abundant food sources resulted in a rapid increase in possum numbers. Currently possums occupy over 90% of New Zealand land area with an estimated 60–70 million possums nation-wide. Possum density estimates range from 1.5 to 25 per hectare and in some areas possum density is 20 times greater than that typically seen in Australia [119].

Seven species of deer were introduced: red deer, sika, white tailed deer, fallow deer (*Dama dama*), elk, sambar deer (*Rusa unicolor*), and rusa deer (*Rusa timorensis*). These deer species were introduced at various times between 1864 and 1907 for recreational hunting purposes. By the middle of the 20th century, red deer numbers had climbed to such levels that they were considered nuisance pests. Farming of red deer began in the 1970s when wild deer were captured to establish breeding herds [119].

*Mycobacterium bovis* was likely introduced to New Zealand with the importation of cattle in the 19th century. By the early 20th century, tuberculosis was recognized as a serious animal and human health problem. Tuberculosis was first diagnosed in farmed deer in 1978 and subsequently spread by movement of untested farmed deer and capture of infected wild deer. The first reported case of tuberculosis in a wild possum in New Zealand was in 1967 [121]. However, the susceptibility of brushtail possums to infection with *M. bovis* had been determined much earlier [122]. Epidemiological evidence links possum tuberculosis and tuberculosis in cattle [123]. Tuberculosis has never been identified in possums in Australia, the original source of New Zealand's possums; therefore, it is likely that possums in New Zealand acquired *M. bovis* from other animals, most likely cattle.

### 4.1. Pathology

*Mycobacterium bovis* infection in possums is typically a fulminating, rapidly fatal pulmonary infection [124] with cases of self-cure being rare exceptions [125]. After infection by natural transmission, death ensues in 3–8 months [126]. Tuberculous possums often develop disseminated disease, with lungs, mediastinal lymph nodes, axillary lymph nodes, and liver being the most common sites of infection. Infection of the axillary lymph nodes frequently leads to formation of discharging sinuses [127]. Lesions are also seen in the spleen, kidneys, adrenal glands, and bone marrow suggesting generalized hematogenous spread of bacilli [128]. In contrast to lesions in cattle, fibrosis, mineralization, and Langhan's type giant cells are uncommon, while AFB are numerous. These characteristics suggest an ineffective host response to infection, leading to an inability to sequester infection, thereby allowing rapid hematogenous dissemination. In spite of disseminated disease, the clinical appearance, body condition, and behavior remain within normal bounds until the latter stages of disease [128]. In contrast, terminally ill possums show a profound change in behavior. The disseminated nature of the disease, with pulmonary lesions, superficial draining sinuses, and large numbers of AFB, combined with the limited effect on behavior for most of the period of infection, make possums an ideal maintenance host capable of efficient transmission to other susceptible hosts.

### 4.2. Epidemiology

Horizontal transmission between possums is principally by infectious aerosol leading to lower respiratory tract infection. Infected possums shed *M. bovis* primarily in respiratory secretions and exudates from superficial draining sinuses. [129]. There is pseudovertical transmission between mother and offspring by aerosol and limited occurrence of pseudovertical transmission by ingestion from tuberculous mastitis [129]. Aerosol transmission may occur at the time of mating and through the sharing of dens. Direct and indirect transmission may also occur through mutual grooming or from a contaminated environment. In studies using captive possums, den sharing provided the greatest risk of transmission between possums [124]. Den sharing has not been commonly observed in free-living possums; however, sequential den use by different possums has been observed [130] and as *M. bovis* can survive inside possum dens for 7–28 days this mode of transmission is possible [56]. *Mycobacterium bovis* remains viable inside possum carcasses for up to 3 days in summer and 27 days in winter and live possums have been observed interacting with possum carcasses. Consequently, transmission from dead infected to live susceptible possums can occur by ingestion, but this route is unlikely to be a significant mode of transmission [131]. The dynamics of possum to possum transmission of *M. bovis* are complex and may involve individual possum social status. Evidence of this was found in captive possum studies where possums infected by natural transmission were those central and prominent in the social hierarchy. Furthermore, when socially dominant possums were experimentally infected, it resulted in higher levels of disease transmission than when possums lower in the societal structure were experimentally infected [132].

Healthy possums generally avoid contact with cattle and deer [133]. However, terminally ill possums exhibit abnormal behavior such as increased daytime activity, stumbling, rolling, and falling, actions that attract the attention of inquisitive cattle and deer. Studies using sedated possums, to simulate terminally ill possums, demonstrated that both deer and cattle exhibit a profound interest in possums behaving abnormally. Cattle were attracted from as far as 50 m to investigate sedated possums [133]. Both deer and cattle were shown to spend significant amounts of time within a distance compatible with aerosol transmission (approximately 1.5 m) and to even sniff, touch, lick, roll, lift, chew, and kick the possum creating an opportunity for direct transmission [130, 134]. In studies where cattle have been excluded from areas used as dens by tuberculous possums, decreased transmission of *M. bovis* from possums to cattle has been demonstrated. In contrast, where cattle are allowed to graze areas used for dens by tuberculous possums transmission to cattle continues unabated [130].

### 4.3. Disease Control Effort

The core of tuberculosis control in New Zealand has been to conduct both an intensive test and slaughter program in cattle and farmed deer, along with equal emphasis to identify and control infected possum populations. Of the wild animals that have been found infected with *M. bovis,* only possums have been targeted for widespread population control, with the intention of reducing possum density and the probability of intraspecies and interspecies transmission. This level of control has approached eradication of possums in some locations [5]. Limited focal culling of ferrets has also been undertaken. No widespread eradication of a vertebrate host has ever been successful on mainland New Zealand, but it has been achieved on some large offshore islands. Social attitudes towards possums in New Zealand differ from those of other wildlife reservoirs of tuberculosis in other countries. In New Zealand, possums are nonnative, invasive pests that cause widespread ecological damage to New Zealand forests and, therefore; widespread removal of possums is desirable for many reasons apart from tuberculosis control. Possums have had a disastrous impact on native flora and fauna. Every night an estimated 70 million possums consume approximately 21,000 tonnes of green shoots, leaves, and berries. Possums are omnivorous and also consume bird's eggs, chicks, small reptiles, and insects. While browsing in the forest canopy on fruits and flowers, possums are in direct competition with native birds. While on the ground, possums compete with native kiwi for dens and have been seen eating kiwi eggs. Theoretically, widespread removal of possums from New Zealand's ecosystem would be more socially palatable than removal of native wildlife reservoirs of tuberculosis in other countries.

It is clear that the key underlying wildlife reservoir of tuberculosis in New Zealand is the infected possum population [5]. Systematic and widespread control of possum populations using poisons and traps has significantly reduced the risk of transmission of tuberculosis to cattle and deer, and the ecological impact of possums. Early control measures included a bounty system on possums that was minimally effective, as it did not allow for prioritization of control efforts, and although many possums were removed, they were generally not removed from essential tuberculosis control areas. The principle method for controlling infected possum populations across large tracts of land is aerial distribution of baits containing 1080 poison (sodium monofluoracetate). An effective poison, 1080 causes cardiac or respiratory failure in possums. Other poisons that have been used to control possums include brodifacoum, pindone, cyanide, and cholecalciferol. Elimination of tuberculosis from possum populations is based on maintaining possum densities at very low levels for a minimum of 5 years [5]. In areas where 1080 baits have been used temporarily to decrease possum numbers for only a limited period of time, tuberculin reactor rates in cattle herds, and numbers of tuberculous possums initially decreased but returned to elevated levels in 8–10 years, with possum numbers recovering through breeding and immigration from surrounding areas [135, 136]. Long-term (>10 year) maintenance of possum populations below 40% of precontrol densities over widespread areas may be required to affect significant change in cattle tuberculin reactor rates and eradicate tuberculosis from possum populations [137]. Reduction in possum populations in *M. bovis* endemic regions has also reduced prevalence in spillover hosts such as feral pigs (*Sus scrofa*) [11].

### 4.4. Vaccination

Although widespread removal of possums through poisoning may decrease the prevalence of tuberculosis in cattle, complete removal of possums from New Zealand may be impractical. It has been suggested that the most promising option for long-term control of tuberculosis in possums may involve targeted vaccination combined with strategies for limited population control or biological control of possums. *Mycobacterium bovis* BCG induces protection and has been administered to possums by subcutaneous, intranasal, intraduodenal, and oral routes [138–142]. All routes provide evidence of protection against aerosol challenge with virulent *M. bovis*, with vaccinates having reduced disease severity, reduced loss of body weight, fewer lung lesions, and decreased bacterial colonization. Two field studies in free-ranging possums in New Zealand have demonstrated that BCG vaccine is protective and can prevent infection. In the first trial, possums were vaccinated by a combination of intranasal aerosol and conjunctival instillation. Vaccine efficacy was estimated at 69% [143]. In the second trial, using oral vaccination, efficacy was estimated at 95%-96% [144].

There are a number of ways that a vaccine could be delivered to free-ranging wild animals, but oral baits are favored as the most practical and cost-effective method [145]. A lipid-based bait has been developed for oral delivery of BCG. Lipid serves to protect viable BCG from degradation in the stomach, allowing lipolytic enzymes of the small and large intestines to liberate BCG, enhancing uptake by gastrointestinal associated lymphoid tissue (GALT) [146]. BCG vaccine delivered in such a fashion persists in mesenteric lymphoid tissue of the GALT for up to 8 weeks [147]. Possums vaccinated with the lipid formulated BCG shed viable BCG in feces for up to 7 days after vaccination, but always in low numbers (<10^3^/gm feces) [147]. Vaccine persistence and shedding are important to evaluate as nontarget species such as scavengers, predators, and even cattle may be exposed to persistent BCG in tissues, feces, or the environment. Notwithstanding, persistence in host tissue, to some degree, is vital for initiation of protective immune response. When administered as lipid formulated BCG to mice, viable BCG persists in lymphoid tissue for up to 30 weeks after vaccination, compared to survival of only 12 weeks when nonlipid formulated BCG was administered [148]. In experimental vaccination and challenge studies, vaccinates showed decreased weight loss, lower lung-body weight ratio, fewer extrapulmonary lesions, and lower lung and spleen bacterial counts compared to nonvaccinates [149]. BCG is avirulent in all animal species so far tested [150], consequently; the risk from BCG persistence in tissues, or excretion in feces, is of little importance, with the exception of livestock. Exposure to BCG could interfere with current diagnostic testing for *M. bovis* infection, making the differentiation of vaccine-exposed cattle from *M. bovis*-infected animals difficult.

### 4.5. Other Wildlife Species

Other species such as red deer, feral pigs, feral cats (*Felis catus*), ferrets, stoats, goats (*Capra hircus*), rabbits (*Oryctolagus cuniculus*), hares (*Lepus europaeus*), and hedgehogs (*Erinaceus europaeus*) have been found infected with *M. bovis* [11, 136, 151]. In New Zealand, research shows feral pigs to be spillover hosts. Exploiting this observation, feral pigs have been used as sentinels in the surveillance for tuberculosis in possums [11, 152]. In contrast, some high-density populations of feral red deer and ferrets are considered maintenance hosts [5]. Other species are inconsequential in the epidemiology of bovine tuberculosis in New Zealand [5].

## 5. Wild Boar in the Iberian Peninsula (Portugal and Spain)—Management of Wild Populations for Commercial Hunting

Increased interest in commercial hunting of animals such as the Eurasian wild boar has resulted in increased fencing of hunting estates. This in turn is linked with increasing population densities, artificial feeding/watering, and translocations, [153] contributing to the widespread distribution of *M. bovis* infection in wild boar in the southern Iberian Peninsula [154]. The experience in Spain is unique compared to other countries in that the prevalence of *M. bovis* infection in wild boar is 100% in some areas, higher than for any other wild ungulate in the Peninsula, or for any other wildlife reservoir in the world [154, 155]. Remarkably, in some areas, this high prevalence of infection exists even in the absence of confounding anthropogenic factors such as artificial feeding [155]. The overall prevalence in red deer is also high at 14% with some areas reaching 50% prevalence in the Iberian Peninsula [154].

Nation-wide, the first efforts to eliminate bovine tuberculosis in Spain began in 1956 [156]. Financial government assistance in the program began in 1965. Early efforts focused on dairy cattle where the prevalence of bovine tuberculosis was estimated to be 20% [156]. In 1991, the prevalence of bovine tuberculosis in Spain was >10%, the highest in the European Community [7, 157]. By 2005, test and slaughter policies had dramatically reduced the prevalence to approximately 0.3% [7]. Restriction fragment length polymorphism analysis and spoligotyping have shown that many of the strains isolated from wild boar are identical to isolates obtained from cattle in the same region [158]. The exact means of interspecies transmission is unclear; however, it is speculated that wild boar contaminate pastures, feed, and sources of water, and thus transmit disease to cattle. Juvenile wild boar are the dispersing age group [159] and may, therefore, contribute to much of the geographical spread of tuberculosis. Philopatric females associate in matriarchal groups, consisting of dam and female offspring. In this environment, intimate contacts between individuals during social and foraging activities are frequent and facilitate pathogen transmission directly and indirectly. Male boar begin to disperse as they become sexually mature (approx, 11 months of age). Dispersal distance increases with age, peaking at 13 months of age and ceasing at 16 months of age, with some males dispersing more than 50 km [159]. The home ranges of adult male boar overlap, except during breeding season when competition for females intensifies. Increased movement of younger males results from aggressive interactions with older males. Extensive tuberculosis lesions in more than one anatomical region have been found in a high proportion of juvenile wild boar that probably represent the main source of mycobacterial excretion [160].

Similarly, *M. bovis* has been identified in other Iberian wildlife, including red deer, fallow deer, Iberian lynx (*Lynx pardinus*), and hare. Again, transmission between cattle and wildlife is implicated due to similar spoligotype patterns of isolates from both livestock and wildlife [161].

One well-studied region is Doñana Biosphere Reserve, a conservation area that is home to a large number of animal species. Hunting and trapping are not allowed within the reserve; however, cattle have been grazed within the reserve for centuries. The first recognized cases of *M. bovis* infection in wildlife within the reserve date back to 1980s. Cattle densities differ between regions within the reserve, but are known to have been as high as 24 per km^2^ [155]. Tuberculin skin testing of cattle in 2006 revealed a reactor rate of 9.4% [155]. In 2006 and 2007, sampling of wild ungulates in the reserve showed *M. bovis* in 52.4% of samples from wild boar, 27.4% from red deer, and 18.5% of fallow deer [155]. A causal link between wildlife tuberculosis and domestic cattle tuberculosis has been shown as removal of wild boar from this site resulted in a corresponding decrease in cattle tuberculosis. In contrast to New Zealand and Australia, wild boar in the Iberian Peninsula are considered one of the main maintenance host species of *M. bovis *[7] and can maintain disease transmission cycles lasting many years [162]. Moreover, data suggest that significant intraspecies transmission likely occurs during the mating season [162].

### 5.1. Pathology

Lymph nodes, principally the paired mandibular lymph nodes are the most commonly affected tissues (>90%) [160]. Greater than 40% of animals have lesions restricted to the mandibular lymph node. Retropharyngeal and parotid lesions are also seen, but generally not without accompanying lesions in the mandibular lymph node [160]. Lymph node lesions generally contain few AFB [160]. Careful microscopic examination of the lungs revealed pulmonary lesions in 38% of the cases [160]. Larger lesions (i.e., 10 cm to 15 cm) were more common in juveniles and those animals with generalized tuberculosis. In addition to cranial lymph nodes and lungs, microscopic lesions are seen in tonsil of the soft palate and ileocecal valve. Microscopically, granulomas are composed of a mixture of epithelioid macrophages and multinucleated giant cells surrounded by infiltrates of lymphocytes, plasma cells, and macrophages. Larger granulomas can contain central regions of caseonecrotic debris that may be mineralized. Multiple bands of fibrous connective tissue may surround larger granulomas. Larger granulomas with more extensive caseonecrosis and mineralization were more common in young animals. In general, intralesional AFB are low in number with the exception of the lung, in which granulomas can contain high numbers of AFB [160].

Over 50% of wild boar have generalized disease. Common involvement of cranial lymph nodes in many animals combined with large numbers of animals showing generalized disease with pulmonary involvement suggests that both oral and aerosol routes are important means of transmission. Contrastingly, in feral pigs in Australia generalized lesions of tuberculosis were seen in <7% of animals and pulmonary lesions were uncommon, supporting the belief that feral pigs in Australia are dead end hosts and transmission from pig to cattle is improbable [9].

In various surveys an extraordinary proportion of both red deer and wild boar in the Iberian Peninsula have presented with disseminated tuberculosis involving numerous organs [154, 155]. Wild boar routinely scavenge carrion, which may explain most of the deer to boar transmission [153]. Monitoring of deer carcasses with motion-activated infrared digital cameras shows carcasses consumed by wild boar, alone or in large packs consisting of males, females, and piglets [155]. Efficient scavengers consuming highly infectious material create an ideal environment for transmission. Both wild boar and deer are known to congregate at watering and feeding sites, especially in managed estates of private land. The aggregation of boar at watering sites is significantly associated with the presence of tuberculosis in both boar and deer. Indeed, aggregation of boar at feeding sites significantly increases the risk of tuberculosis in deer [153]. Gathering of animals at watering and feeding sites increases inter- and intraspecific contact and results in a more heavily contaminated environment where indirect transmission is more likely to occur. Models have shown that interspecific transmission at watering and feeding sites is more likely to be boar to deer rather than deer to boar [153].

### 5.2. Disease Control Effort

The starting point for wildlife disease control is proper disease monitoring. Basic requirements for such monitoring include (1) ensuring the disease is monitored in the relevant domestic animals; (2) ensuring that background information on wildlife host population ecology is available to maximize the benefits of the monitoring effort; (3) selecting the appropriate wildlife hosts for monitoring; (4) selecting the appropriate methods for diagnosis and for time and space trend analysis; (5) deciding which parameters to target for monitoring; finally (6) establishing a reasonable sampling effort and a suitable sampling stratification to ensure detecting changes over time and changes in response to management actions [163].

Three main options exist for control of *M. bovis* transmission at the wild boar-livestock interface: improved biosafety, population control, and vaccination. The first option is being addressed through applied research on cattle feed and waterhole protection, as well as carrion consumption by wild boar, and the risk of leaving behind hunting remains (i.e., viscera not for human consumption). Regarding the second option, in Spain lethal means of population control other than shooting (e.g., poison) are not allowed [164]. Significantly reducing wild boar density through increased hunting reduces tuberculosis prevalence in wild boar and result in lower incidence of tuberculosis in sympatric ruminants. Nevertheless, other means of population control such as feeding bans or contraception remain to be tested. Additionally, newly developed blood tests may allow test-and-cull schemes for removal of infected boars [165, 166].

### 5.3. Vaccination

The third option is BCG vaccination of wild boar to reduce infection prevalence. In controlled laboratory experiments, oral delivery of baits containing BCG reduced *M. bovis* infection and lesion scores by ≥50% in wild boar piglets. Thoracic lesions that correlated with presence and potential excretion of viable *M. bovis* were reduced by 70%, and the serum antibodies against *M. bovis*, which correlate with lesion scores, were reduced by 79%. At the molecular level, oral BCG immunization of wild boar resulted in upregulation of various immunoregulatory genes that may be associated with a protective response to *M. bovis* infection [167, 168]. Recently, a new heat-inactivated *M. bovis* vaccine showed protection and produced wild boar immune responses similar to those seen in BCG-vaccinated boar, suggesting an alternative to use of live BCG in the field [168].

The effective and efficient field vaccination of wildlife species requires the development of stable, species-specific oral baits as delivery vehicles for use in appropriate baiting strategies. Oral baits suitable to deliver pharmaceuticals to free-living wild boar have been developed, as well as techniques to improve specificity and uptake rate in overabundant wild boar populations [164, 167]. Safety experiments yielded no shedding of BCG in feces, even after oral delivery of high doses [169]. Marker studies revealed no bait uptake by nontarget species [170]. In 2010, the Spanish Ministry of Agriculture listed research on wildlife vaccination as a priority in the tuberculosis control strategy. Controlled field experiments with live BCG and the heat-inactivated *M. bovis* vaccine are scheduled for 2012.

### 5.4. Other Species

Although the single most important *M. bovis* maintenance host in the Iberian Peninsula is the wild boar. In actuality, maintenance of *M. bovis* involves a complex, multihost system in which transmission occurs among multiple wild species (wild boar, red deer, fallow deer, and badgers), cattle, and to a lesser extent livestock such as pigs and goats [171].

## 6. Conclusions

Transmission of *M. bovis* across the wildlife-domestic animal interface represents a significant obstacle to bovine tuberculosis eradication efforts in many countries around the world. In spite of long-standing, expensive, and somewhat successful efforts over many decades, animal health officials have found that traditional test and slaughter methods, the centerpiece of most bovine eradication programs, of limited success when affected cattle herds are surrounded by wildlife infected with *M. bovis*. A few countries have implemented various programs intended to decrease the relevant wildlife population density to such a level that interspecies and intraspecies transmission is virtually extinguished. Another commonly used tool is the creation of physical barriers (e.g., fences, animal housing) between cattle and wildlife to mitigate direct and indirect contact. In some cases this requires changing traditional agricultural practices (e.g., methods or location of livestock feeding) that have existed for decades. Such measures have been moderately successful, but in each case animal health officials have concluded that more tools are needed. Wildlife vaccination may be that much needed tool. Significant research will be required on vaccine delivery, safety, and efficacy. As each country battles a different wildlife host, research on each of the reservoir maintenance hosts will be required, as extrapolation of information from one species to another may not be appropriate nor relevant. As such there is a continued need of knowledge of the biology, immunology, and pathology of tuberculosis in each host. Development of successful mitigation strategies will likely require combined efforts and input from the global tuberculosis research community, federal, and regional policy makers (including both animal health and wildlife disease experts), as well as livestock and natural resources stakeholders.

## References

[1] Jones KE, Patel NG, Levy MA (2008). Global trends in emerging infectious diseases. *Nature*.

[2] Daszak P, Cunningham AA, Hyatt AD (2000). Emerging infectious diseases of wildlife—threats to biodiversity and human health. *Science*.

[3] Daszak P, Cunningham AA, Hyatt AD (2001). Anthropogenic environmental change and the emergence of infectious diseases in wildlife. *Acta Tropica*.

[4] Gortázar C, Ferroglio E, Höfle U, Frölich K, Vicente J (2007). Diseases shared between wildlife and livestock: a European perspective. *European Journal of Wildlife Research*.

[5] Ryan TJ, Livingstone PG, Ramsey DSL (2006). Advances in understanding disease epidemiology and implications for control and eradication of tuberculosis in livestock: the experience from New Zealand. *Veterinary Microbiology*.

[6] Carstensen M, Doncarlos MW (2011). Preventing the establishment of a wildlife disease reservoir: a case study of bovine tuberculosis in wild deer in Minnesota, USA. *Veterinary Medicine International*.

[7] Naranjo V, Gortazar C, Vicente J, de la Fuente J (2008). Evidence of the role of European wild boar as a reservoir of *Mycobacterium tuberculosis* complex. *Veterinary Microbiology*.

[8] Schmitt SM, O’Brien DJ, Bruning-Fann CS, Fitzgerald SD (2002). Bovine tuberculosis in Michigan wildlife and livestock. *Annals of the New York Academy of Sciences*.

[9] Corner LA, Barrett RH, Lepper AW, Lewis V, Pearson CW (1981). A survey of mycobacteriosis of feral pigs in the Northern Territory. *Australian Veterinary Journal*.

[10] McInerney J, Small KJ, Caley P (1995). Prevalence of *Mycobacterium bovis* infection in feral pigs in the Northern Territory. *Australian Veterinary Journal*.

[11] Nugent G, Whitford J, Yockney IJ (2012). Reduced spillover transmission of *Mycobacterium bovis* to feral pigs (Sus scofa) following population control of brushtail possums (*Trichosurus vulpecula*). *Epidemiology and Infection*.

[12] Grange JM (2001). *Mycobacterium bovis* infection in human beings. *Tuberculosis*.

[13] Wigle WD, Ashley MJ, Killough EM, Cosens M (1972). Bovine tuberculosis in humans in Ontario. The epidemiologic features of 31 active cases occurring between 1964 and 1970. *American Review of Respiratory Disease*.

[14] Cousins DV, Dawson DJ (1999). Tuberculosis due to *Mycobacterium bovis* in the Australian population: cases recorded during 1970–1994. *International Journal of Tuberculosis and Lung Disease*.

[15] Ravenel MP (1900). Three cases of tuberculosis of the skin due to inoculation with bovine tubercle bacillus. *Philadelphia Medical Journal*.

[16] Robinson P, Morris D, Antic R (1988). *Mycobacterium bovis* as an occupational hazard in abattoir workers. *Australian and New Zealand Journal of Medicine*.

[17] Cooke MM, Gear AJ, Naidoo A, Collins DM (2002). Accidental *Mycobacterium bovis* infection in a veterinarian. *New Zealand Veterinary Journal*.

[18] Fanning A, Edwards S (1991). *Mycobacterium bovis* infection in human beings in contact with elk (*Cervus elaphus*) in Alberta, Canada. *The Lancet*.

[19] Wilkins MJ, Bartlett PC, Frawley B, O’Brien DJ, Miller CE, Boulton ML (2003). *Mycobacterium bovis* (bovine TB) exposure as a recreational risk for hunters: results of a Michigan Hunter Survey, 2001. *International Journal of Tuberculosis and Lung Disease*.

[20] Wilkins MJ, Meyerson J, Bartlett PC (2008). Human *Mycobacterium bovis* infection and bovine tuberculosis outbreak, Michigan, 1994–2007. *Emerging Infectious Diseases*.

[21] Steele JH, Thoen CO, Steele JH (1995). Regional and country status reports. *Mycobacterium bovis Infection in Animals and Humans*.

[22] Belli LB (1962). Bovine tuberculosis in a white-tailed deer. *Canadian Veterinary Journal*.

[23] Ferris DH, Beamer PD, Alberts JO (1961). Tuberculosis in transported deer. A case report. *Journal of Chronic Diseases*.

[24] Friend GH (1963). Tuberculosis in a wild white-tailed deer. *New York Fish and Game Journal*.

[25] Levine PP (1934). A report on tuberculosis in wild deer. *Cornell Veterinarian*.

[26] Schmitt SM, Fitzgerald SD, Cooley TM (1997). Bovine tuberculosis in free-ranging white-tailed deer from Michigan. *Journal of Wildlife Diseases*.

[27] Frye GH, Thoen CO, Steele JH (1995). Bovine tuberculosis eradication: the program in the United States. *Mycobacterium bovis Infection in Animals and Humans*.

[28] McCarty CW, Miller MW (1998). A versatile model of disease transmission applied to forecasting bovine tuberculosis dynamics in white-tailed deer populations. *Journal of Wildlife Diseases*.

[29] Miller R, Kaneene JB, Fitzgerald SD, Schmitt SM (2003). Evaluation of the influence of supplemental feeding of white-tailed deer (*Odocoileus virginianus*) on the prevalence of bovine tuberculosis in the Michigan wild deer population. *Journal of Wildlife Diseases*.

[30] O’Brien DJ, Schmitt SM, Fierke JS (2002). Epidemiology of *Mycobacterium bovis* in free-ranging white-tailed deer, Michigan, USA, 1995–2000. *Preventive Veterinary Medicine*.

[31] Palmer MV, Waters WR, Whipple DL (2004). Shared feed as a means of deer-to-deer transmission of *Mycobacterium bovis*. *Journal of Wildlife Diseases*.

[32] Whipple DL, Clarke PR, Jarnagin JL, Payeur JB (1997). Restriction fragment length polymorphism analysis of *Mycobacterium bovis* isolates from captive and free-ranging animals. *Journal of Veterinary Diagnostic Investigation*.

[33] Fitzgerald SD, Kaneene JB, Butler KL (2000). Comparison of postmortem techniques for the detection of *Mycobacterium bovis* in white-tailed deer (*Odocoileus virginianus*). *Journal of Veterinary Diagnostic Investigation*.

[34] Palmer MV, Whipple DL, Payeur JB (2000). Naturally occurring tuberculosis in white-tailed deer. *Journal of the American Veterinary Medical Association*.

[35] Beatson NS, Brown RD (1985). Tuberculosis in red deer in New Zealand. *Biology of Deer Production.*.

[36] Lugton IW, Wilson PR, Morris RS, Nugent G (1998). Epidemiology and pathogenesis of *Mycobacterium bovis* infection of red deer (*Cervus elaphus*) in New Zealand. *New Zealand Veterinary Journal*.

[37] Robinson RC, Phillips PH, Stevens G, Storm PA (1989). An outbreak of *Mycobacterium bovis* infection in fallow deer (*Dama dama*). *Australian veterinary journal*.

[38] Whiting TL, Tessaro SV (1994). An abattoir study of tuberculosis in a herd of farmed elk. *Canadian Veterinary Journal*.

[39] Thompson AK, Samuel MD, Van Deelen TR (2008). Alternative feeding strategies and potential disease transmission in Wisconsin white-tailed deer. *Journal of Wildlife Management*.

[40] Dunkley L, Cattet RL (2003). *A Comprehensive Review of Ecological and Human Social Effects of Artificial Feeding and Baiting of Wildlife*.

[41] Kaneene JB, Miller R, De Kantor IN, Thoen CO (2010). Tuberculosis in wild animals. *International Journal of Tuberculosis and Lung Disease*.

[42] Bruning-Fann CS, Schmitt SM, Fitzgerald SD (2001). Bovine tuberculosis in free-ranging carnivores from Michigan. *Journal of Wildlife Diseases*.

[43] Bruning-Fann CS, Schmitt SM, Fitzgerald SD (1998). *Mycobacterium bovis* in coyotes from Michigan. *Journal of Wildlife Diseases*.

[44] Kaneene JB, Bruning-Fann CS, Dunn J (2002). Epidemiologic investigation of *Mycobacterium bovis* in a population of cats. *American Journal of Veterinary Research*.

[45] O’Brien DJ, Schmitt SM, Fitzgerald SD, Berry DE, Hickling GJ (2006). Managing the wildlife reservoir of *Mycobacterium bovis*: the Michigan, USA, experience. *Veterinary Microbiology*.

[46] Palmer MV, Waters WR, Whipple DL (2002). Susceptibility of raccoons (*Procyon lotor*) to infection with *Mycobacterium bovis*. *Journal of Wildlife Diseases*.

[47] Wells WF (1948). On the mechanics of droplet nuclei infection: I. Apparatus for the quantitative study of droplet nuclei infection of animals. *American Journal of Epidemiology*.

[48] Wells WF, Ratcliffe HL, Crumb C (1948). On the mechanics of droplet nuclei infection: II. Quantitative experimental air-borxr tuberculosis in rabbits. *American Journal of Epidemiology*.

[49] Blanchong JA, Scribner KT, Kravchenko AN, Winterstein SR (2007). TB-infected deer are more closely related than non-infected deer. *Biology Letters*.

[50] Hill J (2005). *Wildlife-cattle interactions in Northern Michigan: implications for the transmission of bovine tuberculosis*.

[51] Palmer MV, Whipple DL, Olsen SC (1999). Development of a model of natural infection with *Mycobacterium bovis* in white-tailed deer. *Journal of Wildlife Diseases*.

[52] Palmer MV, Whipple DL, Waters WR (2001). Experimental deer-to-deer transmission of *Mycobacterium bovis*. *American Journal of Veterinary Research*.

[53] Palmer MV, Waters WR, Whipple DL (2004). Investigation of the transmission of *Mycobacterium bovis* from deer to cattle through indirect contact. *American Journal of Veterinary Research*.

[54] Duffield BJ, Young DA (1984). Survival of *Mycobacterium bovis* in defined environmental conditions. *Veterinary Microbiology*.

[55] Fine AE, Bolin CA, Gardiner JC (2011). A study of the persistence of *Mycobacterium bovis* in the environment under natural weather conditions in Michigan, USA. *Veterinary Medicine International*.

[56] Jackson R, de Lisle GW, Morris RS (1995). A study of the environmental survival of *Mycobacterium bovis* on a farm in New Zealand. *New Zealand Veterinary Journal*.

[57] Palmer MV, Whipple DL (2006). Survival of *Mycobacterium bovis* on feedstuffs commonly used as supplemental feed for white-tailed deer (*Odocoileus virginianus*). *Journal of Wildlife Diseases*.

[58] Tanner M, Michel AL (1999). Investigation of the viability of M. bovis under different environmental conditions in the Kruger National Park. *Onderstepoort Journal of Veterinary Research*.

[59] O’Brien DJ, Fitzgerald SD, Lyon TJ (2001). Tuberculous lesions in free-ranging white-tailed deer in Michigan. *Journal of Wildlife Diseases*.

[60] Palmer MV, Waters WR, Whipple DL (2002). Milk containing *Mycobacterium bovis* as a source of infection for white-tailed deer fawns (*Odocoileus virginianus*). *Tuberculosis*.

[61] Shilang Z, Shanzhi W (1985). Prevention of tuberculosis in sika deer (*Cervus nippon*). *Biology of Deer Production*.

[62] Griffin JFT, Mackintosh CG, Slobbe L, Thomson AJ, Buchan GS (1999). Vaccine protocols to optimise the protective efficacy of BCG. *Tubercle and Lung Disease*.

[63] Griffin JFT, MacKintosh CG, Rodgers CR (2006). Factors influencing the protective efficacy of a BCG homologous prime-boost vaccination regime against tuberculosis. *Vaccine*.

[64] Nol P, Palmer MV, Waters WR (2008). Efficacy of oral and parenteral routes of *Mycobacterium bovis* bacille Calmette-Guerin vaccination against experimental bovine tuberculosis in white-tailed deer (*Odocoileus virginianus*): a feasibility study. *Journal of Wildlife Diseases*.

[65] Palmer MV, Thacker TC, Waters WR (2007). Vaccination of white-tailed deer (*Odocoileus virginianus*) with *Mycobacterium bovis* bacillus Calmette Guerín. *Vaccine*.

[66] Palmer MV, Thacker TC, Waters WR (2009). Vaccination with *Mycobacterium bovis* BCG strains Danish and Pasteur in white-tailed deer (*Odocoileus virginianus*) experimentally challenged with *Mycobacterium bovis*. *Zoonoses and Public Health*.

[67] Palmer MV, Thacker TC, Waters WR, Robbe-Austerman S, Lebepe-Mazur SM, Harris NB (2010). Persistence of *Mycobacterium bovis* bacillus calmette-guérin in white-tailed deer (*Odocoileus virginianus*) after oral or parenteral vaccination. *Zoonoses and Public Health*.

[68] Palmer MV, Thacker TC, Waters WR (2007). Vaccination of white-tailed deer (*Odocoileus virginianus*) with *Mycobacterium bovis* bacillus Calmette Guerín. *Vaccine*.

[69] Bouvier G (1963). Transmision possible de la Tuberculose et de la Brucellose du giber a lhomme et aux animaux domestiques et sauvages. *Bulletin—Office International Epizooties*.

[70] Muirhead RH, Gallagher J, Burn KJ (1974). Tuberculosis in wild badgers in Gloucestershire: epidemiology. *Veterinary Record*.

[71] Noonan NL, Sheane WD, Harper LR, Ryan PJ (1975). Wild-life as a possible reservoir of bovine tuberculosis. *Irish Veterinary Journal*.

[72] DEFRA Great Britain regional and county bovine TB statistics.

[73] Rogers LM, Forrester GJ, Wilson GJ, Yarnell RW, Cheeseman CL (2003). The role of setts in badger (*Meles meles*) group size, breeding success and status of TB (*Mycobacterium bovis*). *Journal of Zoology*.

[74] Delahay RJ, Brown JA, Mallinson PJ (2000). The use of marked bait in studies of the territorial organization of the European Badger (*Meles meles*). *Mammal Review*.

[75] Rogers LM, Delahay R, Cheeseman CL, Langton S, Smith GC, Clifton-Hadley RS (1998). Movement of badgers (*Meles meles*) in a high-density population: individual, population and disease effects. *Proceedings of the Royal Society B*.

[76] Sleeman DP, Mulcahy MF (2005). Loss of territoriality in a local badger (*Meles meles*) population at Kilmurry, Co. Cork, Ireland. *Irish Naturalists’ Journal*.

[77] Kruuk H (1989). *The Social Badger: Ecology and Behaviour of a Group-Living Carnivore (*Meles meles*)*.

[78] Cresswell WJ, Harris S (1988). Foraging behaviour and home-range utilization in a suburban badger (*Meles meles*) population. *Mammal Review*.

[79] O'Corry-Crowe G, Eves J, Hayden TJ, Hayden TJ (1993). Sett distribution, territory size and population density of badgers (*Meles meles*) in east Offlay. *The Badger*.

[80] Cleary GP, Corner LAL, O’Keeffe J, Marples NM (2009). The diet of the badger *Meles meles* in the Republic of Ireland. *Mammalian Biology*.

[81] Sleeman DP, Davenport J, More SJ (2009). How many Eurasian badgers *Meles meles* L. are there in the Republic of Ireland?. *European Journal of Wildlife Research*.

[82] Cheeseman CL, Wilesmith JW, Stuart FA (1989). Tuberculosis: the disease and its epidemiology in the badger, a review. *Epidemiology and Infection*.

[83] Corner LAL, Costello E, Lesellier S, O’Meara D, Gormley E (2008). Experimental tuberculosis in the European badger (*Meles meles*) after endobronchial inoculation with *Mycobacterium bovis*: II. Progression of infection. *Research in Veterinary Science*.

[84] Gallagher J, Clifton-Hadley RS (2000). Tuberculosis in badgers; a review of the disease and its significance for other animals. *Research in Veterinary Science*.

[85] Corner LAL, O'Meara D, Costello E The distribution of *Mycobacterium bovis* infection in naturally infected badgers.

[86] Gallagher J, Muirhead RH, Burn KJ (1976). Tuberculosis in wild badgers (*Meles meles*) in Gloucestershire: pathology. *Veterinary Record*.

[87] Gavier-Widen D, Chambers MA, Palmer N, Newell DG, Hewinson RG (2001). Pathology of natural *Mycobacterium bovis* infection in European badgers (*Meles meles*) and its relationship with bacterial excretion. *Veterinary Record*.

[88] Corner LAL, Murphy D, Gormley E (2011). *Mycobacterium bovis* infection in the Eurasian badger (*Meles meles*): the disease, pathogenesis, epidemiology and control. *Journal of Comparative Pathology*.

[89] Dungworth DL (1993). The respiratory system. *Pathology of Domestic Animals*.

[90] Little TWA, Naylor PF, Wilesmith JW (1982). Laboratory study of *Mycobacterium bovis* infection in badgers and calves. *Veterinary Record*.

[91] Hutchings MR, Harris S (1997). Effects of farm management practices on cattle grazing behaviour and the potential for transmission of bovine tuberculosis from badgers to cattle. *Veterinary Journal*.

[92] Garnett BT, Delahay RJ, Roper TJ (2002). Use of cattle farm resources by badgers (*Meles meles*) and risk of bovine tuberculosis (*Mycobacterium bovis*) transmission to cattle. *Proceedings of the Royal Society B*.

[93] Scantlebury M, Hutchings MR, Allcroft DJ, Harris S (2004). Risk of disease from wildlife reservoirs: badgers, cattle, and bovine tuberculosis. *Journal of Dairy Science*.

[94] Krebs JR, Anderson RM, Clutton-Brock T (1998). Badgers and bovine TB: conflicts between conservation and health. *Science*.

[95] Murphy D, Gormley E, Collins DM (2011). Tuberculosis in cattle herds are sentinels for *Mycobacterium bovis* infection in European badgers (*Meles meles*): the Irish Greenfield Study. *Veterinary Microbiology*.

[96] Smith RMM, Drobniewski F, Gibson A (2004). *Mycobacterium bovis* Infection, United Kingdom. *Emerging Infectious Diseases*.

[97] Woodroffe R, Donnelly CA, Jenkins HE (2006). Culling and cattle controls influence tuberculosis risk for badgers. *Proceedings of the National Academy of Sciences of the United States of America*.

[98] Donnelly CA, Woodroffe R, Cox DR (2003). Impact of localized badger culling on tuberculosis incidence in British cattle. *Nature*.

[99] Wilson GJ, Carter SP, Delahay RJ (2011). Advances and prospects for management of TB transmission between badgers and cattle. *Veterinary Microbiology*.

[100] Bourne FJ, Donnelly CA, Cox DR (2006). TB policy and the badger culling trials. *Veterinary Record*.

[101] Godfrey CJ, Curnow RN, Dye C (2005). *Independent Scientific Review of the Randomized Badger Culling Trial and Association Epidemiological Research*.

[102] More SJ, Clegg TA, McGrath G, Collins JD, Corner LAL, Gormley E (2007). Does reactive badger culling lead to an increase in tuberculosis in cattle?. *Veterinary Record*.

[103] Griffin JM, Williams DH, Kelly GE (2005). The impact of badger removal on the control of tuberculosis in cattle herds in Ireland. *Preventive Veterinary Medicine*.

[104] Costello E, O’Grady D, Flynn O (1999). Study of restriction fragment length polymorphism analysis and spoligotyping for epidemiological investigation of *Mycobacterium bovis* infection. *Journal of Clinical Microbiology*.

[105] O’Keeffe JJ (2006). Description of a medium term national strategy toward eradication of tuberculosis in cattle in Ireland. *Biennial Report, 2004-2005*.

[106] Gormley E, Collins JD (2000). The development of wildlife control strategies for eradication of tuberculosis in cattle in Ireland. *Tubercle and Lung Disease*.

[107] Ó Máirtín D, Williams DH, Griffin JM, Dolan LA, Eves JA (1998). The effect of a badger removal programme on the incidence of tuberculosis in an Irish cattle population. *Preventive Veterinary Medicine*.

[108] Olea-Popelka FJ, Fitzgerald P, White P (2009). Targeted badger removal and the subsequent risk of bovine tuberculosis in cattle herds in county Laois, Ireland. *Preventive Veterinary Medicine*.

[109] Vial F, Donnelly CA (2012). Localized reactive badger culling increases risk of bovine tuberculosis in nearby cattle herds. *Biology Letters*.

[110] Böhm M, Hutchings MR, White PCL (2009). Contact networks in a wildlife-livestock host community: identifying high-risk individuals in the transmission of bovine TB among badgers and cattle. *PLoS ONE*.

[111] Corner LAL, Costello E, Lesellier S, O’Meara D, Gormley E (2008). Vaccination of European badgers (*Meles meles*) with BCG by the subcutaneous and mucosal routes induces protective immunity against endobronchial challenge with *Mycobacterium bovis*. *Tuberculosis*.

[112] Stuart FA, Mahmood MF, Stanford JL (1988). Development of diagnostic tests, and vaccination against, tuberculosis in badgers. *Mammal Review*.

[113] Chambers MA, Rogers F, Delahay RJ (2011). Bacillus Calmette-Guérin vaccination reduces the severity and progression of tuberculosis in badgers. *Proceedings of the Royal Society B*.

[114] Corner LAL, Costello E, O’Meara D (2010). Oral vaccination of badgers (*Meles meles*) with BCG and protective immunity against endobronchial challenge with *Mycobacterium bovis*. *Vaccine*.

[115] Delahay RJ, Wilson GJ, Smith GC, Cheeseman CL (2003). Vaccinating badgers (*Meles meles*) against *Mycobacterium bovis*: the ecological considerations. *Veterinary Journal*.

[116] Aldwell FE, Keen DL, Parlane NA, Skinner MA, De Lisle GW, Buddle BM (2003). Oral vaccination with *Mycobacterium bovis* BCG in a lipid formulation induces resistance to pulmonary tuberculosis in brushtail possums. *Vaccine*.

[117] Aznar I, McGrath G, Murphy D (2011). Trial design to estimate the effect of vaccination on tuberculosis incidence in badgers. *Veterinary Microbiology*.

[118] Delahay RJ, Smith GC, Barlow AM (2007). Bovine tuberculosis infection in wild mammals in the South-West region of England: a survey of prevalence and a semi-quantitative assessment of the relative risks to cattle. *Veterinary Journal*.

[119] O’Neil BD, Pharo HJ (1995). The control of bovine tuberculosis in New Zealand. *New Zealand Veterinary Journal*.

[120] Wodzicki K, Wright S (1984). Introduced birds and mammals in New Zealand and their effect on the environment. *Tuatara*.

[121] Ekdahl MO, Smith BL, Money DF (1970). Tuberculosis in some wild and feral animals in New Zealand. *New Zealand Veterinary Journal*.

[122] Bolliger A, Bolliger W (1948). Experimental transmission of tuberculosis to *Trichosurus vulpecula*. *The Australian Journal of Science*.

[123] Collins DM, Gabric DM, de Lisle GW (1988). Typing of *Mycobacterium bovis* isolates from cattle and other animals in the same locality. *New Zealand Veterinary Journal*.

[124] Corner LAL, Buddle BM, Morris RS (2003). Experimental infection of brushtail possums (*Trichosurus vulpecula*) with *Mycobacterium bovis* by conjunctival instillation. *Veterinary Journal*.

[125] Corner LAL, Norton S (2003). Resolution of *Mycobacterium bovis* infection in wild brushtail possums (*Trichosurus vulpecula*). *New Zealand Veterinary Journal*.

[126] Norton S, Corner LAL, Morris RS (2005). Ranging behaviour and duration of survival of wild brushtail possums (*Trichosurus vulpecula*) infected with *Mycobacterium bovis*. *New Zealand Veterinary Journal*.

[127] Cooke MM, Jackson R, Coleman JD (1995). Naturally occurring tuberculosis caused by Mycobacterium bovis in brushtail possums (*Trichosurus vulpecula*): II. Pathology. *New Zealand Veterinary Journal*.

[128] Jackson R, Cooke MM, Coleman JD (1995). Naturally occurring tuberculosis caused by *Mycobacterium bovis* in brushtail possums (*Trichosurus vulpecula*): I. An epidemiological analysis of lesion distribution. *New Zealand Veterinary Journal*.

[129] Jackson R, Cooke MM, Coleman JD (1995). Naturally occurring tuberculosis caused by *Mycobacterium bovis* in brushtail possums (*Trichosurus vulpecula*): III. Routes of infection and excretion. *New Zealand Veterinary Journal*.

[130] Paterson BM, Morris RS, Weston J (1995). Foraging and denning patterns of brushtail possums, and their possible relationship to contact with cattle and the transmission of bovine tuberculosis. *New Zealand Veterinary Journal*.

[131] Barron MC, Pech RP, Whitford J (2011). Longevity of *Mycobacterium bovis* in brushtail possum (*Trichosurus vulpecula*) carcasses, and contact rates between possums and carcasses. *New Zealand Veterinary Journal*.

[132] Corner LAL, Pfeiffer DU, Morris RS (2003). Social-network analysis of *Mycobacterium bovis* transmission among captive brushtail possums (*Trichosurus vulpecula*). *Preventive Veterinary Medicine*.

[133] Paterson BM, Morris RS (1995). Interactions between beef cattle and simulated tuberculous possums on pasture. *New Zealand Veterinary Journal*.

[134] Sauter CM, Morris RS (1995). Behavioural studies on the potential for direct transmission of tuberculosis from feral ferrets (*Mustela furo*) and possums (*Trichosurus vulpecula*) to farmed livestock. *New Zealand Veterinary Journal*.

[135] Barlow ND (1991). A spatially aggregated disease/host model for bovine Tb in New Zealand possum populations. *Journal of Applied Ecology*.

[136] Tweddle NE, Livingstone P (1994). Bovine tuberculosis control and eradication programs in Australia and New Zealand. *Veterinary Microbiology*.

[137] Caley P, Hickling GJ, Cowan PE, Pfeiffer DU (1999). Effects of sustained control of brushtail possums on levels of *Mycobacterium bovis* infection in cattle and brushtail possum populations from Hohotaka, New Zealand. *New Zealand Veterinary Journal*.

[138] Aldwell FE, Keen DL, Stent VC (1995). Route of BCG administration in possums affects protection against bovine tuberculosis. *New Zealand Veterinary Journal*.

[139] Aldwell FE, Pfeffer A, DeLisle GW (1995). Effectiveness of BCG vaccination in protecting possums against bovine tuberculosis. *Research in Veterinary Science*.

[140] Buddle BM, Aldwell FE, Keen DL, Parlane NA, Yates G, De Lisle GW (1997). Intraduodenal vaccination of brushtail possums with bacille Calmette-Guérin enhances immune responses and protection against *Mycobacterium bovis* infection. *International Journal of Tuberculosis and Lung Disease*.

[141] Corner LAL, Buddle BM, Pfeiffer DU, Morris RS (2001). Aerosol vaccination of the brushtail possum (*Trichosurus vulpecula*) with bacille Calmette-Guérin: the duration of protection. *Veterinary Microbiology*.

[142] Corner LAL, Norton S, Buddle BM, Morris RS (2002). The efficacy of bacille Calmette-Guérin vaccine in wild brushtail possums (*Trichosurus vulpecula*). *Research in Veterinary Science*.

[143] Corner LAL, Buddle BM, Pfeiffer DU, Morris RS (2002). Vaccination of the brushtail possum (*Trichosurus vulpecula*) against *Mycobacterium bovis* infection with bacille Calmette-Guérin: the response to multiple doses. *Veterinary Microbiology*.

[144] Tompkins DM, Ramsey DSL, Cross ML, Aldwell FE, De Lisle GW, Buddle BM (2009). Oral vaccination reduces the incidence of tuberculosis in free-living brushtail possums. *Proceedings of the Royal Society B*.

[145] Cross ML, Buddle BM, Aldwell FE (2007). The potential of oral vaccines for disease control in wildlife species. *Veterinary Journal*.

[146] Aldwell FE, Tucker IG, De Lisle GW, Buddle BM (2003). Oral delivery of *Mycobacterium bovis* BCG in a lipid formulation induces resistance to pulmonary tuberculosis in mice. *Infection and Immunity*.

[147] Wedlock DN, Aldwell FE, Keen D, Skinner MA, Buddle BM (2005). Oral vaccination of brushtail possums (*Trichosurus vulpecula*) with BCG: immune responses, persistence of BCG in lymphoid organs and excretion in faeces. *New Zealand Veterinary Journal*.

[148] Aldwell FE, Cross ML, Fitzpatrick CE, Lambeth MR, De Lisle GW, Buddle BM (2006). Oral delivery of lipid-encapsulated *Mycobacterium bovis* BCG extends survival of the bacillus in vivo and induces a long-term protective immune response against tuberculosis. *Vaccine*.

[149] Cross ML, Henderson RJ, Lambeth MR, Buddle BM, Aldwell FE (2009). Lipid-formulated bcg as an oral-bait vaccine for tuberculosis: vaccine stability, efficacy, and palatability to brushtail possums (*Trichosurus vulpecula*) in New Zealand. *Journal of Wildlife Diseases*.

[150] Murphy D, Corner LAL, Gormley E (2008). Adverse reactions to *Mycobacterium bovis* bacille Calmette-Guérin (BCG) vaccination against tuberculosis in humans, veterinary animals and wildlife species. *Tuberculosis*.

[151] Jackson R (2002). The role of wildlife in *Mycobacterium bovis* infection of livestock in New Zealand. *New Zealand Veterinary Journal*.

[152] Nugent G, Whitford J, Young N (2002). Use of released pigs as sentinels for *Mycobacterium bovis*. *Journal of Wildlife Diseases*.

[153] Vicente J, Höfle U, Garrido JM (2007). Risk factors associated with the prevalence of tuberculosis-like lesions in fenced wild boar and red deer in south central Spain. *Veterinary Research*.

[154] Vicente J, Höfle U, Garrido JM (2006). Wild boar and red deer display high prevalences of tuberculosis-like lesions in Spain. *Veterinary Research*.

[155] Gortázar C, Torres MJ, Vicente J (2008). Bovine tuberculosis in Doñana Biosphere Reserve: the role of wild ungulates as disease reservoirs in the last Iberian lynx strongholds. *PLoS ONE*.

[156] Allepuz A, Casal J, Napp S (2011). Analysis of the spatial variation of Bovine tuberculosis disease risk in Spain (2006–2009). *Preventive Veterinary Medicine*.

[157] Caffrey JP (1994). Status of bovine tuberculosis eradication programmes in Europe. *Veterinary Microbiology*.

[158] Serraino A, Marchetti G, Sanguinetti V (1999). Monitoring of transmission of tuberculosis between wild boars and cattle: genotypical analysis of strains by molecular epidemiology techniques. *Journal of Clinical Microbiology*.

[159] Truvé J, Lemel J (2003). Timing and distance of natal dispersal for wild boar (Sus scrofa) in Sweden. *Wildlife Biology*.

[160] Martín-Hernando MP, Höfle U, Vicente J (2007). Lesions associated with *Mycobacterium* tuberculosis complex infection in the European wild boar. *Tuberculosis*.

[161] Aranaz A, De Juan L, Montero N (2004). Bovine tuberculosis (*Mycobacterium bovis*) in wildlife in Spain. *Journal of Clinical Microbiology*.

[162] Parra A, Larrasa J, García A, Alonso JM, Hermoso De Mendoza J (2005). Molecular epidemiology of bovine tuberculosis in wild animals in Spain: a first approach to risk factor analysis. *Veterinary Microbiology*.

[163] Boadella M, Gortazar C, Acevedo P (2011). Six recommendations for improving monitoring of diseases shared with wildlife: examples regarding mycobacterial infections in Spain. *European Journal of Wildlife Research*.

[164] Ballesteros C, Carrasco-García R, Vicente J (2009). Selective piglet feeders improve age-related bait specificity and uptake rate in overabundant Eurasian wild boar populations. *Wildlife Research*.

[165] Boadella M, Lyashchenko K, Greenwald R (2011). Serologic tests for detecting antibodies against *Mycobacterium bovis* and *Mycobacterium avium* subspecies paratuberculosis in Eurasian wild boar (*Sus scrofa scrofa*). *Journal of Veterinary Diagnostic Investigation*.

[166] Lyashchenko KP, Greenwald R, Esfandiari J (2008). Animal-side serologic assay for rapid detection of *Mycobacterium bovis* infection in multiple species of free-ranging wildlife. *Veterinary Microbiology*.

[167] Ballesteros C, Garrido JM, Vicente J (2009). First data on Eurasian wild boar response to oral immunization with BCG and challenge with a *Mycobacterium bovis* field strain. *Vaccine*.

[168] Garrido JM, Sevilla IA, Beltran-Beck B (2011). Protection against tuberculosis in Eurasian wild boar vaccinated with heatinactivated *Mycobacterium bovis*. *PLoS ONE*.

[169] Ballesteros C, Gortázar C, Canales M (2009). Evaluation of baits for oral vaccination of European wild boar piglets. *Research in Veterinary Science*.

[170] Ballesteros C, Vicente J, Carrasco-García R, Mateo R, de la Fuente J, Gortázar C (2011). Specificity and success of oral-bait delivery to Eurasian wild boar in Mediterranean woodland habitats. *European Journal of Wildlife Research*.

[171] Gortazar C, Delahay RJ, McDonald RA The status of tuberculosis in European wild mammals.

